# Toward understanding sexual immune dimorphism in humans

**DOI:** 10.3389/fimmu.2025.1570565

**Published:** 2025-06-20

**Authors:** Tomasz J. Nowak, Michael P. Muehlenbein

**Affiliations:** Laboratory for Evolutionary Medicine and One Health, Department of Anthropology, Baylor University, Waco, TX, United States

**Keywords:** sexual immune dimorphism, immune-endocrine interactions, regulation of immunity, life history theory, estrogen, testosterone, X-chromosome inactivation, pathogen avoidance

## Abstract

Sexual immune dimorphism refers to the distinct differences in immune responses between males and females, influenced by genetic, hormonal, developmental, social, and behavioral factors. These differences, shaped by evolutionary pressures, manifest in varied susceptibilities to infectious and autoimmune diseases, as well as differences in vaccine responses and disease outcomes. Females generally exhibit stronger immune responses than males, which confer protection against infections but also lead to a higher prevalence of autoimmune diseases. Hormones such as estrogen, progesterone, and testosterone play pivotal roles in modulating these responses. Estrogen enhances immune activation, promoting inflammation and increasing autoimmune susceptibility, while testosterone exerts primarily immunosuppressive effects, reducing autoimmune risks but heightening infection susceptibility. Genetic factors, including X-linked immune-related genes and cellular mosaicism, further contribute to the observed dimorphism, as do epigenetic mechanisms that modulate immune gene expression. From an evolutionary perspective, life history theory explains these differences as the result of trade-offs between reproductive strategies and immune function, with females prioritizing robust immunity for offspring survival and males balancing immune investment with reproductive fitness. Behavioral factors, such as pathogen avoidance and risk-taking, add complexity to the dimorphism. This review adopts a narrative format intentionally designed to provide a cohesive conceptual synthesis of major mechanisms underlying sexual immune dimorphism. While acknowledging the complexity and breadth of this topic, we explicitly focus on integrating hormonal, genetic/epigenetic, behavioral, and evolutionary contexts. By examining the interplay of these factors, the review provides a foundation for understanding the biological underpinnings and evolutionary context of immune differences between sexes.

## Introduction

Sexual immune dimorphism refers to the distinct differences in immune system functions and responses between males and females, driven by genetic, hormonal, developmental, behavioral, and other factors. These differences are rooted in evolutionary pressures that shape distinct reproductive strategies and life history traits for each sex (1). The recognition of sexual dimorphism in immunity emerged from observations of differential disease incidence and severity between males and females. Early studies noted that females typically have ‘stronger’ immune responses than males, leading to lower likelihoods of pathogen infection rates but higher incidence of autoimmune conditions (2). Estrogen and progesterone in females, and androgens like testosterone in males, play significant roles in modulating immune responses. For example, testosterone levels in males are often directly associated with higher susceptibility to disease from pathogens and lowered inflammatory responses – conversely, females typically exhibit stronger immune responses, partly due to the immunomodulatory effects of estrogens, which are crucial for maintaining reproductive health and successful pregnancy (3, 4). Several genes that are crucial for immune function are located on the X chromosome providing another mechanism for stronger immune responses in females compared to males (5). Behavioral and social factors such as propensity for risk taking or pathogen disgust are also important contributors to sex immune dimorphism (6).

The immune system is a set of behavioral and physiological responses and barriers that, in general, protect against infection, respond to allergens, and control unregulated cellular growth. The first major component of this system is innate immunity that defends against pathogens through a collection of nonspecific responses. These include physiological agents such as macrophages, neutrophils, natural killer (NK) cells, and the complement system—a group of proteins that enhances the immune system’s ability to combat infections. Anatomical barriers like skin or epithelium are considered parts of innate immunity. Another essential part of innate, nonspecific immunity is health and sickness behaviors (7), including avoidance of sources of contamination and illness (disgust) and lethargy to conserve energy while ill. Adaptive immunity is a component responsible for specific responses to pathogens and, unlike innate immunity, is acquired predominantly through previous exposure. Responsible for these responses include lymphocytes, both B cells and T cells; both types can produce a response to current infection and memory cells that are used in response to subsequent exposure to a similar pathogen. B cells (or plasma cells) are responsible for the production of immunoglobulins (Ig) in different variations (IgA, IgD, IgE, IgG, IgM). T cells can be divided generally into cytotoxic T cells (CD8+) and helper T cells (CD4+) which can be further subdivided into T helper (Th) 1, Th2, T regulatory cells (T_reg_) and others that are responsible for different responses (8). The reader is referred to various excellent texts and reviews of human immunology for more detail outside the present brief review.

Immune responses are modulated in part by the actions of hormones produced by the endocrine system, including sex steroids like estrogen and testosterone. This relationship highlights the interconnectedness of these systems, where fluctuations in hormone levels can modulate immune activity, influencing susceptibility to infections and the overall strength of immune defenses. The endocrinology of the reproductive system encompasses a complex network of hormones and tissues that regulate reproductive functions and behaviors. Among reproductive age human females, the cyclic production of hormones regulates the menstrual cycle, including follicular development, ovulation, and the maintenance of the endometrium. Estrogen and progesterone play critical roles in pregnancy, lactation, and development of secondary sexual characteristics (9). In human males, testosterone is essential for spermatogenesis, the development of male secondary sexual characteristics, and the maintenance of libido (10). The reproductive endocrine system also involves feedback mechanisms where sex steroids influence the release of gonadotropin-releasing hormone, luteinizing hormone, and follicle-stimulating hormone (11). The adrenal glands further contribute to the production of androgens – this intricate endocrine interplay ensures the proper functioning of reproductive processes and the expression of reproductive behaviors, with significant implications for fecundity and overall health (11).

One of the first connections between endocrinology and immunology demonstrated at the end of the 19^th^ century when it was noted that castrated rabbits developed a more prominent thymus than non-castrated rabbits (12). For several decades, slow integration of endocrinology and immunology took place, resulting in a review of early findings in the *“Network of Immune-Neuroendocrine Interactions”* (13). It was proposed that the immune system is interconnected with neuroendocrine structures via afferent-efferent pathways so that signals can flow to and from the immune system, affecting hypothalamic activity and hormone levels. These interactions were demonstrated to be crucial for regulating immune responses as well as other physiological and developmental processes. Additional work demonstrated estrogens and dihydrotestosterone receptors present on thymic tissue, and that estrogen replacement therapy after castration can reverse thymus enlargement, providing further evidence for communication between endocrine and immune tissues (14–16). This work spurred early investigations into immunological differences between individuals, sexes, populations, and species, and contemporary research continues to explore immune-endocrine interactions, especially in the context of autoimmune diseases and differential susceptibility to infection. Studies have further detailed the complex interactions between the hypothalamic-pituitary-adrenal axis and immune responses, underscoring the intricate balance maintained by hormones and immune factors in health and disease (17).

Understanding sexual immune dimorphism in humans has broad implications for public health, medicine, and scientific research. Recognizing differences in immune responses between males and females can lead to more personalized medical treatments, improve the efficacy of therapies, and minimize adverse effects by tailoring treatments based on sex-specific immune profiles. Different susceptibilities and responses to infectious diseases, autoimmune disorders, and cancers between males and females should be considered when designing healthcare interventions. Many recent works focus on sex-based differences in efficacy and safety of vaccines and specific differences in response to vaccination (18). Understanding sexual immune dimorphism also provides insights into the evolutionary pressures and processes that have shaped immune system development, contributing to our understanding of human evolutionary biology. The primary objective of the present review is to provide a brief overview of the current understanding of sexual immune dimorphism in humans, outlining key differences in immune responses between males and females. This review will also explore and explain the potential roles of some hormones and genetic/epigenetic factors as well as behaviors that likely contribute to these sexual differences, specifically providing evolutionary and ecological context for why natural selection has resulted in human sexual immune dimorphism to enhance evolutionary fitness. Scope is limited to only the most major and well understood mechanisms, as a comprehensive review warrants, at minimum, an edited volume. Furthermore, immunological differences are explicitly detailed between sexes that result from genetic/physiological differences, to the exclusion of conversations about gender as determined by choice.

This narrative review serves as an accessible conceptual framework integrating diverse mechanisms of sexual immune dimorphism. We recognize this approach inherently involves selective coverage of mechanisms that are most supported by current evidence. It is explicitly structured to foster cross-disciplinary dialogue, encourage hypothesis development, and stimulate further focused systematic studies.

## Sexual immune dimorphism

### Evolutionary perspectives

Life history theory posits that each organism must judiciously allocate its finite resources among competing factors, including survival, reproduction, growth, and maintenance (19). These resources should be distributed in manners that maximize survivorship and reproductive success. Given the limited nature of available resources, trade-offs between these functions are inevitable. These trade-offs should differ between the sexes because of pronounced differences in reproductive effort and associated costs of reproduction (20). Natural selection has produced different strategies by which males and females ensure genetic contributions to future generations. By the nature of their biology, mammalian females have a higher investment in offspring in terms of gestation and postnatal care (21). These greater investments in reproduction are selection pressures that favor stronger immune systems to ensure survival and reproductive success (22). Robust immune responses in females would help protect both the mother and the developing offspring from pathogens. Male reproductive biology and behaviors are under different selection pressures that have favored, for example, higher testosterone levels that can exert modulatory costs of immunity (23, 24). Trade-offs between investment in reproduction (e.g., competition, mating effort, muscle anabolism) and immune function could lead to lower immune responses in males compared to females (25).

Sexual selection has influenced observed differences in immune functions between males and females. Females tend to invest more in traits that enhance maintenance and survivorship, which in turn support their reproductive success over time. This investment includes stronger immune defenses to protect themselves and their offspring, and this evolutionary pressure led to more robust immune responses in females compared to males (25). In contrast to females, male reproductive effort includes (among other things) investment in secondary sexual characteristics that can facilitate competition for mates, including signals of fitness (26). High levels of testosterone may enhance these competitive traits but at potential costs to immunity. Testosterone’s immunomodulatory actions are complex and often suppressive, so it is possible and probable that those males with higher testosterone levels may, under different circumstances, produce lowered immune responses in a trade-off with reproductive success against survivorship and maintenance (24).

Sexual conflict arises from differing reproductive strategies between males and females (27). This conflict drives the evolution of sex-specific traits, including immune responses. The immune traits and states optimized for one sex may not be optimal for the other, leading to antagonistic selection on shared immune genes. Expression of those genes can be up- or down-regulated by the actions of sex hormones (28), further leading to observed outcomes in clinical data. Females experience significant changes in sex hormones throughout their lives that can result in patterns of immunity not observable in males. For example, females experience significant immune modulation during pregnancy in order to tolerate the fetus, influencing overall immune function and contributing to sex differences in disease susceptibility and progression (29). In fact, the immune system in females has likely evolved partially to compensate for the unique demands of pregnancy (ibid). The Pregnancy Compensation Hypothesis (PCH) suggests that sex-specific immune modulation evolved to facilitate the survival of the pregnant female in the presence of an invasive placenta and immunologically-challenging pregnancy. The PCH further suggests that evolution of the eutherian placenta exerted significant sex-specific selection pressures on immune function to tolerate fetal antigens while still defending against pathogens (30). Immunity during pregnancy is regulated by reproductive hormones and gene expression on the sex chromosomes, and the maternal immune system must balance tolerance to the genetically distinct fetus with protection against infections, further leading to sex-specific immune responses. Indeed, the immune system undergoes significant modulation during pregnancy as the result of hormonal changes. Studies show that pregnancy-related hormones like estriol can alleviate symptoms of autoimmune diseases such as multiple sclerosis and rheumatoid arthritis (31).

Hormonal fluctuations during the menstrual cycle may have evolved to balance reproductive success and immune defense. The concept of a “choosy uterus” suggests that menstruation might be a byproduct of an evolutionary strategy to optimize reproductive success by cyclically modulating immunity (32). The uterus undergoes cyclical changes in preparation for pregnancy, becoming highly selective about which embryos it allows to implant. This selectivity ensures that only the healthiest embryos, which are most likely to result in a successful pregnancy, are supported.

Immune responses vary throughout the menstrual cycle. During the luteal phase, after ovulation, progesterone levels rise along with a correlated increase in regulatory T cells that constrain immune responses (33). This suppression is crucial for allowing a genetically distinct embryo (which could be interpreted as a foreign body) to implant and develop without being neutralized by the maternal immune system. Conversely, the follicular phase is associated with a more active immune environment, which could help with eliminating any pathogens and non-viable cells (33).

Disgust and pathogen avoidance are also components of the immune system that should display sexual dimorphism. The emotion of disgust is thought to have evolved primarily as a defense mechanism to protect from pathogens and contaminants [however more subtypes of disgust have been described (34)]. The expression of disgust coincides with critical periods when humans are vulnerable to pathogen ingestion, such as the post-weaning period in early childhood (34). Human females report higher levels of disgust than males across various domains, including job selection, mate choice, food aversions, and psychological disorders (6). Females also display stronger physiological responses, such as skin conductivity, to disgust stimuli (35). The evolutionary advantage for females to have higher disgust sensitivity, especially in terms of pathogen disgust, is hypothesized to be closely linked to their roles in reproduction and caregiving; heightened sensitivity to disgust in females likely evolved as a mechanism to help to avoid pathogens. This is crucial because females, particularly during pregnancy, lactation, and caregiving, are in closer contact with offspring who have underdeveloped immune systems and are more vulnerable to infections. This is encapsulated in the “mothers matter more” hypothesis which suggests that, because mothers are more critical for the survival and health of offspring, there were stronger selection pressures on females to avoid harm and pathogens (6, 36).

Disgust sensitivity, particularly towards pathogens, undergoes significant changes during pregnancy (37). This phenomenon has been studied to understand its implications for maternal and fetal health, primarily focusing on how increased sensitivity may serve as a protective mechanism during this vulnerable period. Research indicates that disgust sensitivity, especially in relation to food, is elevated during the first trimester of pregnancy. This heightened sensitivity correlates with the period of greatest immunosuppression and vulnerability to pathogens for both the mother and fetus (37). Rising progesterone levels during pregnancy are associated with increased disgust sensitivity. This hormone, which suppresses aspects of immunity to protect the fetus, simultaneously stimulates behavioral immunity by increasing proneness to disgust, thereby aiding in pathogen avoidance (38). Pregnancy-related immunosuppression is also linked to changes in dietary behavior, notably increased aversions to meat, which is more likely to carry pathogens. This avoidance behavior likely evolved to protect both mother and fetus from infections during periods of heightened vulnerability (39).

These theories and hypotheses illustrate the complex interplay between reproductive strategies, immune function, and evolutionary pressures. They highlight the necessity of considering sex-specific and life history traits to understand immune responses and reproductive strategies in humans and other species. This understanding not only enriches our knowledge of evolutionary biology and behavioral ecology but also has important implications for health and disease management.

### Potential mechanisms

#### Hormones

There are several direct explanations of sexual immune dimorphism that are not mutually exclusive and have additive or multiplicative effects. Sex hormones play crucial roles in orchestrating human sexual development, sexuality, reproduction, and immunity. They influence life history transitions such as puberty and childbirth and respond to contextual factors like energy flux (caloric intake and expenditure) and physical/psychological stress (40). Hormone patterns, particularly those related to the menstrual cycle, play significant roles in reproductive behaviors as well as physiology (41). Because sexual dimorphism is necessary to maximize evolutionary fitness, disrupting sex hormones levels could be detrimental for developing or maintaining appropriate immunity. For example, endocrine disrupting chemicals (EDCs) such as bisphenols, phthalates, and parabens affect the development, function, and lifespan of various immune cells, including monocytes, neutrophils, lymphocytes, and dendritic cells (42). EDCs interfere with cytokine, immunoglobulin, and inflammatory mediator synthesis, affecting immune cell activation and survival (43), potentially resulting in immunodeficiency or hyperreactivity.

Immune cells express receptors for many hormones, including estrogens, progesterone, and androgens (44), and these receptors mediate the effects of sex steroids on immune cell activation, lifespan, and function. Sex steroid receptors in immune cells influence sexual immune dimorphism through several specific mechanisms. Estrogen receptors α (ERα) and β (ERβ) are the two main types of estrogen receptors found in immune cells, and modulate immune responses by upregulating pro-inflammatory cytokines and increasing the activity of T cells, B cells, and macrophages (45, 46). ERα is particularly involved in stimulating immune responses, while ERβ serves more regulatory roles (47). Higher estrogen levels in females are linked to increased prevalence of autoimmune diseases due, in part, to stimulation of autoantibody production and autoreactive B cell survival (48). Estrogen can also upregulate Bcl-2, a protein that inhibits apoptosis, thus aiding in the survival of autoreactive B cells and potentially leading to autoimmunity (49). Recent studies highlight the significance of the membrane-bound G protein-coupled estrogen receptor (GPER) in modulating immune responses (47). GPER is expressed in peripheral B and T lymphocytes, monocytes, eosinophils, neutrophils, NK cells, and dendritic cells – these patterns of expression suggest that GPER has a broad role in modulating immune functions across different immune cell phenotypes (50). GPER activation can affect various immune functions, including the enhancement of chemotaxis in eosinophils, important for allergic inflammation (47). Additionally, estrogens can facilitate hematopoietic stem cell (HSC) self-renewal in pregnant female mice, confirming the presence of ERs on HSCs and their contribution to sexual immune dimorphism (although general HSC functions are thought to be comparable between sexes) (51). Estradiol also positively regulates human hematopoiesis *in vitro* (52).

Estrogen can influence immunity via interactions throughout the hypothalamic–pituitary–adrenal (HPA) axis. Although some mechanisms remain speculative, the HPA axis appears to regulate several proinflammatory pathways in addition to producing immunosuppressive glucocorticoids (53). Glucocorticoids downregulate activation of leucocytes, cytokine production, and the sensitivity to cytokines, suppress Th1 cell production, and increase apoptosis of some T cells and eosinophils (54). In animal models, infusion of estrogen directly into the brain can increase corticosterone response (55). Alternatively, estradiol appears to downregulate the HPA axis in experiments involving rhesus macaques (56). In humans, females in the luteal phase have a more robust cortisol response to the trier social stress test (TSST) than women in the early follicular phase (when estrogen levels are expected to be lower) (57). Thus, the HPA axis has a potentially significant but short effect on human immunity likely mediated in part by estrogen upregulating and downregulating different parts of the axis. It is currently unknown if this mechanism is a significant factor contributing to sexual immune dimorphism either in human or non-human animals.

A complete review of all hormonal pathways is beyond the scope of this manuscript; however, we note that estrogen plays a complex role in immune regulation, exhibiting both immunostimulatory and immunosuppressive effects depending on concentration, life stage, and disease context. At physiological levels, estrogen enhances innate immunity by increasing type I interferon production, stimulating macrophage activity, and promoting leukocyte proliferation, thereby bolstering immune surveillance (58). It also modulates toll-like receptor expression, further enhancing immune responses (59). However, at supra-physiological levels, estrogen exerts an immunosuppressive effect by reducing pro-inflammatory cytokine production, downregulating CD8+ T cells, and inhibiting IL-2 receptor expression, ultimately limiting T-cell proliferation and dampening cellular immunity (60). This immunosuppressive role is particularly evident in pregnancy, where high estrogen levels shift immune responses toward Th2 dominance, reducing autoimmune symptoms but increasing susceptibility to infections (61). Estrogen’s effects also vary with life stage, as neonatal exposure to estrogenic compounds can cause permanent immune dysfunction, while adult exposure tends to have reversible effects (62). In disease contexts, estrogen has been found to play a protective role in multiple sclerosis by reducing inflammation, yet it exacerbates lupus by increasing autoantibody production (63). Furthermore, exposure to environmental estrogens, such as endocrine disruptors, may alter lymphoid organ development and immune cell function, potentially leading to immune dysregulation (64). These findings highlight the dynamic and context-dependent nature of estrogen’s influence on immune function, providing critical insights into sex-based differences in immunity and informing potential therapeutic strategies for immune-related conditions.

Progesterone, a key female sex hormone, also plays a significant role in modulating immune responses, likely contributing to several observed differences between male and female immune function. The immunomodulatory effects of progesterone also differ from those of estrogens and androgens. At low physiological levels, progesterone seems to enhance pathways necessary for the development of autoimmune diseases like systemic lupus erythematosus, while at higher pregnancy levels suppress disease activity in rheumatoid arthritis and multiple sclerosis by inhibiting Th1 and Th17 pathways and inducing anti-inflammatory molecules (65). Progesterone can inhibit pro-inflammatory responses by suppressing cytokine production by dendritic cells (DCs), reducing their ability to stimulate T cells, and downregulating co-stimulatory molecules (66). Progesterone promotes maternal-fetal tolerance by reducing T-cell polyfunctionality and inducing a specific cytokine profile that skews towards anti-inflammatory responses (67). Progesterone also delays neutrophil apoptosis, which can alter inflammatory responses and enhance the production of reactive oxygen intermediates, contributing to a stronger immune response (68). It inhibits the production of pro-inflammatory cytokines like IL-1β, TNFα, and IL-6 in macrophages by modulating Toll-like receptor (TLR) signaling and enhancing the expression of suppressor of cytokine signaling (SOCS1) (69). Progesterone also promotes the differentiation of naïve T cells into regulatory T cells and suppresses their differentiation into Th17 cells, which are associated with inflammation. This modulation is important for maintaining immune tolerance and preventing autoimmunity (70).

Androgens such as testosterone play crucial roles in mediating immune responses and contribute significantly to the observed differences in immune functions between males and females. These hormones generally have immunosuppressive effects, influencing both innate and adaptive immunity (24). They typically suppress immune functions by decreasing antibody production, T cell proliferation, and natural killer cell cytotoxicity. They also stimulate the production of anti-inflammatory cytokines (71). This suppression leads to a lower incidence of autoimmune diseases in males compared with females but increases susceptibility to infections and certain cancers (72). Androgens directly modulate the development and function of immune cells, impacting innate immune responses. For instance, androgens are required for the generation and proper function of neutrophils and play a role in macrophage recruitment and pro-inflammatory cytokine production (73). Androgens suppress both T-cell and B-cell immune responses, and the effects are more pronounced during the developmental stages of these cells in the thymus and bone marrow rather than on mature effector cells (74). Androgen deprivation therapy (ADT) in males can lead to an increase in T-cell levels and enhance T-cell responses, suggesting a potential strategy for boosting immune function in certain therapeutic contexts (75).

#### Genetics

Genetic differences between males and females originate from the sex chromosomes. During early stages of female embryogenesis, one X chromosome is inactivated to prevent the overproduction of proteins in females. However, some regions escape this inactivation and lead to cellular mosaicism. Since each X-chromosome is inherited from a different parent, females have two genetically distinct types of cells (76) and can produce more diverse immune responses. Some X-lined genes that lack their Y counterparts can also escape inactivation. As a result, some females will express both copies of X-linked genes while other females will express only one copy. Moreover, since males have only one copy of X-linked genes, they are prone to mutations in some immune-related genes that reside on the X chromosome. This phenomenon causes numerous X-linked primary immunodeficiencies. For example, the mutation in the *CD40L* gene (resulting in X-linked hyper-IgM syndrome or CD40L deficiency) tends not to affect females, while in males it causes lower levels of IgG, IgE, and IgA (and often higher levels of IgM), neutropenia, impaired NK and T cell cytotoxicity, and makes them suffer from recurrent and opportunistic infections (77).

Cellular mosaicism can be responsible for dysregulation of self-tolerance and higher autoimmune disease prevalence in females. Females can possess two distinct dendritic cell populations that either express paternal or maternal X-linked self-antigens, leading to a negative selection of thymocytes if the mosaicism is a random process. However, if it is skewed toward one X-linked chromosome, females would produce mostly one set of self-antigens on DCs and tolerate T-cells skewed toward the second set of self-antigens, leading to autoimmunity (78–80). Another hypothesis postulates that females’ autoimmunity can be caused by reactivation of X-linked immune-related genes from an inactive chromosome or overexpression of such genes from the active X chromosome (81). More on connections between cellular mosaicism and autoimmunity can be found in (76).

Epigenetics play a significant role in sexual immune dimorphism by influencing how sex hormones like estrogen, progesterone, and testosterone modulate immune expression. Studies have identified a sex-specific transcriptome and methylome in various immune cells, independent of X-chromosome inactivation; an integrative analysis of the methylome and transcriptome in circulating immune cells reveals sex-specific differentially-methylated regions (82). These regions are associated with immune cell-specific gene expression profiles, suggesting that sexual dimorphism also occurs at the epigenetic level. Ligand-bound nuclear hormone receptors (such as estrogen, progesterone, and androgen receptors) can interact with co-regulators to alter chromatin structure and histone tail modifications, facilitating or inhibiting gene transcription (4). Periods of hormonal change, such as puberty, pregnancy, menopause, and hormone therapy (both menopausal and gender-affirming), are associated with significant epigenetic reprogramming (83). These changes can affect immune system functions and the expression of immune-related genes. During puberty, there is an increase in sex hormones—testosterone in males and estrogen and progesterone in females. These hormones induce epigenetic changes, such as DNA methylation and histone modifications. Genes associated with immune pathways undergo differential methylation during puberty, which affects immune responses (84). Pregnancy involves significant increases in hormones like human chorionic gonadotropin (hCG), progesterone, and estrogen which drive extensive epigenetic remodeling in immune cells. For instance, hCG can influence histone modifications (85), while estrogen modifies DNA methylation patterns (86). These changes can alter the transcriptome of immune cells and promote an anti-inflammatory state necessary for fetal tolerance.

### Infectious disease differences

Infectious diseases exhibit notable disparities in incidence and prevalence between sexes, influenced by a complex interplay of physiological, genetic, behavioral, and hormonal factors (**Table 1**). These differences not only affect susceptibility to infections but also shape physiological responses, influence the effectiveness of the immune system, and determine clinical outcomes. Sexual immune dimorphism in relation to some of these pathogenic infections and some potential causes are discussed below.

**Table 1:** Sex-based differences in prevalence and incidence of selected infectious diseases.

Disease	Males	Females	References
Influenza	Higher hospitalization rates in males under 18 and over 40 years; males in the same age group less affected.	Higher morbidity and mortality during reproductive years; stronger humoral immune response to vaccines but more adverse reactions.	87–89
COVID-19	Higher severity linked to low testosterone levels and lifestyle factors like smoking.	More likely to adhere to preventative measures; higher stress levels motivating guideline adherence.	90–92
Pneumonia (CAP)	47%-98% higher incidence in males; attributed to lifestyle factors like smoking and comorbidities.	Lower incidence overall; likely influenced by fewer comorbidities and lifestyle factors.	93, 94
Tuberculosis (TB)	Male-to-female ratio approximately 1.7:1; higher prevalence linked to genetic and occupational factors.	Lower prevalence overall; stronger immune responses likely due to physiological differences.	95, 96
Urinary Tract Infections (UTIs)	Less common; often associated with complications like obstructions or prostate issues.	Lifetime prevalence of 50-60%; higher risk due to anatomy, sexual activity, and hygiene practices	97, 98

This table highlights the comparative prevalence and incidence rates of various infectious diseases in males and females, detailing the underlying biological and behavioral factors contributing to these disparities.

Multiple studies illustrate that influenza infections tend to affect females on average more severely than males with higher morbidity and mortality rates in females during reproductive years (88, 89). A study in Hong Kong identified that boys under 18 years of age had higher hospitalization rates for influenza compared with girls, with the male-to-female ratio of excess hospitalization rates ranging from 1.1 to 2.4 (87). In adults younger than 40 years of age, hospitalization rates were lower for males than females, except for influenza A(H3N2); for adults over 40 years of age, males generally had higher hospitalization rates for influenza (ibid).

Incidence rates of influenza infection among pregnant females vary, but are generally higher compared to non-pregnant females, especially during influenza pandemics (99). Pregnant females are considered a high-risk group for severe influenza outcomes. Females generally mount stronger humoral immune responses to influenza vaccines but also experience more adverse reactions than males (89). This is due to higher levels of proinflammatory cytokines and chemokines in females. During pregnancy, the immune system shifts towards a more anti-inflammatory state to tolerate the fetus (as discussed above), reducing the body’s ability to combat viral infections like influenza (29).

Studies demonstrate that serum testosterone concentrations during acute COVID-19 disease in males are inversely proportional to inflammatory cytokines and illness severity (90). Testosterone levels could be also used as a prognostic value of outcomes related to COVID-19 disease (100). Behavioral difference also likely influences incidence of respiratory diseases. For example, males have higher smoking rates both globally and across demographic groups (91), and smoking exacerbates the severity of COVID-19 by compromising immune responses and increasing risk of severe outcomes, including hospitalization and death (101, 102).

Females appear more likely to adhere to social distancing and quarantine guidelines, reducing their risk of infection and spread. For example, one review identified that females, older individuals, and those who more likely trust government sources of information are more likely to adhere to COVID-19 guidelines (92). Research also indicates that females experienced higher levels of stress during the COVID-19 pandemic, which may have motivated them to adhere more strictly to guidelines to mitigate perceived risks. During the early months of the pandemic, females were more likely to engage in CDC guideline-adherent behaviors, particularly social distancing and hygiene practices (103).

Pneumonia is more common in males than females. A study in Spain from 2016–2019 found that males had a 47% higher adjusted incidence of community-acquired pneumonia (CAP) than females, while hospital-acquired pneumonia was 98% higher in males compared to females. This study reported an incidence rate of 384.5 to 449.8 cases per 100,000 population in males and 244.9 to 301.2 cases per 100,000 in females (93). Similarly, in a study conducted in Ohio, U.S., the incidence of CAP requiring hospitalization was higher in males (291.4 per 100,000) compared to females (244.8 per 100,000), with incidence rates increasing significantly with age (104). In another U.S. study, the incidence of CAP requiring hospitalization was higher in males than in females with the highest rates among older adults (105).

Apart from biological differences between males and females that affect responses to pathogens, studies indicate the significant impacts of comorbidities and social factors. Conditions such as chronic obstructive pulmonary disease (COPD), diabetes, and heart diseases are more prevalent in males, increasing their risk for CAP. Conditions like COPD significantly increase the risk of CAP in seniors, particularly among males (94). Lifestyle choices such as smoking and alcohol consumption, which are more common in males, also contribute to a higher incidence of CAP. Smoking damages the respiratory tract and impairs immune function, increasing susceptibility to infections (106). Males are also more likely to be employed in occupations with higher exposure to respiratory hazards, such as construction, manufacturing, and mining. These exposures can increase the risk of developing CAP due to inhalation of harmful chemicals and particulate matter (107).

The incidence of tuberculosis (TB) varies between males and females, with males generally experiencing higher rates of infection, prevalence, and mortality compared to females. This gender disparity in TB incidence is influenced by a combination of biological, epidemiological, and socio-economic factors. Globally, the male-to-female ratio for TB case notifications is approximately 1.7 (108). A systematic review and meta-analysis found that TB prevalence was significantly higher among males than females in low- and middle-income countries; the male-to-female prevalence ratios were 2.21 for bacteriologically-positive TB and 2.51 for smear-positive TB (95).

Males have smaller B cell follicles in the lungs compared, in general, to females, with resultant less effective immune responses against *Mycobacterium tuberculosis*, and this impaired follicle formation in the lungs is associated with accelerated disease progression in males (96). Genetic polymorphisms in Toll-like receptor (TLR) genes, specifically TLR7 and TLR8, are associated with increased susceptibility to TB in males. These polymorphisms result in less effective phagocytosis of *Mycobacterium tuberculosis* and impaired TLR signaling, contributing to higher infection rates in males (109). Other factors that explain higher prevalence of TB in males is engagement in social behaviors like smoking and alcohol consumption that are significant determinants of higher TB incidence (110). It has further been suggested that traditional masculine norms, such as prioritizing work and avoiding seeking help, delay males from accessing healthcare for TB, and a delay can result in advanced disease stages by the time of diagnosis (111). Certain occupations predominantly held by males, such as mining, construction, and transportation, also expose them to higher risks of TB due to poor working conditions and prolonged exposure to dust and silica (112).

In contrast with the above examples, urinary tract infection (UTI) is more common in females than males, with a lifetime incidence of 50−60% in adult females – prevalence increases with age, and is approximately double in females over age 65 compared with the overall female population (98). Although less common, UTIs in males are more likely to be complicated and associated with underlying pathologies such as urinary tract obstructions or prostate issues (97). Females are more likely to experience recurrent UTIs due to anatomy, hygiene practices, sexual activity, and contraception use: the female urethra is shorter than the male urethra and so bacteria have a shorter distance to travel to reach the bladder (113); proximity to the anus increases the likelihood of bacteria spreading to urinary tract (97); and sexual intercourse can introduce bacteria into the urinary tract (114).

### Autoimmune disease differences

Sexual dimorphism in immune responses between males and females results in difference in autoimmune disease prevalence and incidence (**Table 2**). These differences are influenced by, among other things, genetic/epigenetic and hormonal factors which have significant implications for understanding and treating these diseases. As already discussed above, estrogen and progesterone in females, and testosterone in males, play significant roles in modulating immune responses. With several genes associated with immune function located on the X chromosome, females may also have a higher risk due to X-linked genes escaping inactivation (123). Most autoimmune diseases show female-bias in terms of prevalence and more severe clinical symptoms.

**Table 2:** Comparative analysis of prevalence and incidence rates of selected autoimmune diseases between females and males, highlighting significant sex differences.

Autoimmune Disease	Frequency in Females	Frequency in Males	Female-to-Male Ratio	References
Type 1 Diabetes (T1D)	Lower age-adjusted prevalence than in males in multiple studies.	Higher incidence (e.g., male-to-female ratio ~1.8:1 in Swedish and 1.32:1 in U.S. studies).	~1:1.3 to ~1:1.8 (incidence)	115, 116
Rheumatoid Arthritis (RA)	Higher incidence and prevalence (e.g., 65.7 per 100,000 annually in Rochester, Minnesota).	Lower incidence and prevalence (e.g., 28.1 per 100,000 annually in males).	~3:1	117, 118
Psoriasis	Similar prevalence (~2.5-3.2%).	Similar prevalence (~2.8-3.2%).	~1:1	119, 120
Multiple Sclerosis (MS)	Higher prevalence and incidence (e.g., 450.1 per 100,000 in U.S. females).	Lower prevalence and incidence (e.g., 159.7 per 100,000 in U.S. males).	~2.8:1	121
Systemic Lupus Erythematosus (SLE)	Higher prevalence and incidence (e.g., ~45.4 per 100,000 in U.K. females).	Lower prevalence and incidence (e.g., ~3.7 per 100,000 in U.K. males).	~9:1	122

Data reflect findings from multiple population-based studies.

Some of the most common autoimmune diseases are reviewed below to further illustrate sex differences in underlying immunity that may explain differences in disease frequency and outcome. First, type-1 diabetes (T1D) is one of the most common and well-studied chronic diseases, demonstrating characteristic dimorphism between men and women. In a Swedish sample, incidence of T1D is higher in males than in females across different age groups, with a male-to-female ratio of approximately 1.8:1 (115). Similarly, in a Lithuanian sample, age-adjusted prevalence of T1D is higher in males compared to females in the 15–34 year age group; the overall mean increase in T1D prevalence is 1.25% annually, with a higher increase in males (124). A longitudinal study in the United States confirms higher incidence of T1D in males than females, with a male-to-female incidence rate ratio of 1.32. This trend is evident from age 10 and persists throughout adulthood (116).

Rheumatoid arthritis (RA) is a chronic inflammatory autoimmune disease that affects joints and other tissues. RA is more common in females than males, with a ratio of approximately 3:1. One study reports an average annual incidence rate of 65.7 per 100,000 population for females and 28.1 per 100,000 for males in Rochester, Minnesota (117). Another study finds the overall annual incidence of RA to be 53.1 per 100,000 in females and 27.7 per 100,000 in males in Olmsted County, Minnesota (118). This trend is sustained across different countries such as Norway (125), Italy (126), and South Korea (127). Females also tend to have higher disease activity and experience more severe symptoms (128, 129), and sex hormones such as estrogen and testosterone likely play significant roles in RA pathogenesis. As previously described, estrogen tends to enhance immune responses, which may contribute to the higher prevalence and severity of RA in females. Conversely, testosterone has immunosuppressive effects, which may provide some protective effect in males (130). Females tend to have a higher prevalence of Th17 cells and a lower prevalence of T regulatory cells, leading to a more pronounced inflammatory response. This imbalance in the Th17/Treg axis seems to contribute to the higher disease activity in females with RA (131).

Psoriasis is a chronic inflammatory skin condition that can significantly affect quality of life, including aspects of sexual health and psychological well-being. The prevalence of psoriasis is generally similar between males and females, but studies show differences in severity and body distribution of lesions; females tend to have more severe disease and higher prevalence of psoriasis on the scalp and genital regions (132, 133). Studies in Denmark, United States, and Italy show the prevalence of psoriasis to be around 2.5-3.2% for females and 2.8-3.2% for males (119, 120, 134).

Hormonal differences between sexes may contribute to the observed dimorphism in psoriasis. Female sex hormones, particularly during pregnancy and menopause, can influence the course and severity of the disease. Estrogen has immunomodulatory effects that may exacerbate or alleviate psoriasis symptoms (4), downregulating production of pro-inflammatory chemokines and cytokines such as CXCL8, CXCL10, and IL-12 while enhancing the production of anti-inflammatory IL-10 (135). Progesterone also contributes to skin health by preventing epidermal atrophy and enhancing collagen synthesis, which can mitigate some of the skin damage experienced in psoriasis (135). In male patients, higher levels of estradiol correlate inversely with the Psoriasis Area and Severity Index (PASI), suggesting increased estradiol levels may reduce disease severity (136). Estrogens suppress the production of psoriasis-related cytokines such as IL-1β and IL-23 from neutrophils and dendritic cells, respectively, and this suppression likely helps mitigate the inflammatory response associated with psoriasis (137). Hormonal changes during puberty, menstrual cycles, pregnancy, and menopause may influence psoriasis. For example, high estrogen levels during pregnancy often lead to improvement in psoriasis, while post-partum (when estrogen levels drop) the condition frequently worsens (138).

Testosterone may help reduce the severity of psoriasis. Studies indicate that increased testosterone levels are associated with reduced inflammatory responses in psoriasis (130). Treatments targeting hormone receptors, such as tamoxifen, induce remission in some cases of psoriasis, indicating the potential therapeutic benefits of modulating hormonal pathways (139). Sex hormones, particularly estrogen and testosterone, significantly impact the progression and severity of psoriasis. Targeted hormone therapies that differ across life stages offer potential avenues for managing psoriasis more effectively.

Multiple sclerosis (MS) is a chronic autoimmune disease affecting the central nervous system, characterized by inflammation, demyelination, and neurodegeneration (140). Sex differences in MS include higher prevalence in females, who tend to experience more frequent relapses, while males often face a more progressive and severe disease course. Females are up to three times more likely to develop MS than males (141). In 2019, the estimated prevalence of MS in the U.S. adult population was 309.2 per 100,000; the prevalence was 450.1 per 100,000 in females and 159.7 per 100,000 in males, with a sex ratio of 2.8:1 (121). An additional U.S.-based study, using electronic health data, shows females ages 50 to 69 years have the highest prevalence, over 600 per 100,000 (142), suggesting an influence of menopause. In other MS studies females consistently exhibit higher prevalence than males, with notable differences in some regions: in the United Kingdom, the prevalence in 2014 was 285.8 per 100,000 in females versus 113.1 in males, with incidence rates of 11.52 and 4.84 per 100,000/year, respectively (143). Similar trends have been observed in Italy, Norway, Canada, and Sweden, where females have higher prevalence and incidence rates, demonstrating a clear sex disparity in MS across diverse geographical locations (144–147). However, males often experience a more severe disease progression and worse clinical outcomes (141).

Sex hormones, such as estrogen and testosterone, appear to play crucial roles in modulating MS immune responses and disease activity. With its anti-inflammatory effects, estrogen may contribute to the milder disease course observed during pregnancy (148). Estrogen and progesterone enhance the function of regulatory T cells, which are crucial in controlling inflammation and autoimmunity in MS (149).

Systemic lupus erythematosus (SLE) is a chronic autoimmune disease characterized by inflammation and damage to various tissues, including the skin, joints, kidneys, and nervous system. SLE is more common in females than males, with a sex ratio of approximately 9:1. This disparity is observed globally and across various age groups, particularly during reproductive years (150, 151). One study in United Kingdom identified prevalence of SLE to be 3.7 per 100,000 for males and 45.4 per 100,000 for females (122). More recently, in Germany, SLE incidence was estimated around 0.9 per 100,000 person-years for males and 1.9 per 100,000 people-years for females; the highest incidence in males was 2.2 per 100,000 at age 65-70, while the highest incidence in females 3.6 per 100,000 at age 20-25 (152). These differences suggest a potentially important role of sex hormones in SLE: estrogen enhances the survival of autoreactive B cells and increases the production of autoantibodies, contributing to the higher prevalence of SLE in females (48). Hormonal fluctuations during puberty, pregnancy, and menopause significantly affect SLE activity. The female-to-male ratio of SLE increases after puberty, suggesting a role of sex hormones, particularly estrogens, in disease susceptibility (153). Pregnancy-induced hormonal changes have a potential to increase the likelihood of disease activity (154). For males, testosterone seems to protective against SLE by reducing the severity of immune responses; males with SLE often have lower levels of testosterone which are typically associated with increased disease activity and severity (155).

Sexual dimorphism in autoimmune diseases is a complex phenomenon influenced by genetic/epigenetic and hormonal factors. These differences manifest in the prevalence, incidence, and severity of autoimmune diseases between males and females. Estrogen and progesterone in females and testosterone in males play crucial roles in modulating immune responses, contributing to these differences. Although testosterone is present in females and estrogen in males, their levels are generally too low to exert the same immunomodulatory effects observed in the opposite sex. Type 1 diabetes, rheumatoid arthritis, multiple sclerosis, and systemic lupus erythematosus all exhibit notable sex-based disparities in their clinical presentation and progression, and with exception of type 1 diabetes they show female-bias (156).

#### Primary immunodeficiencies

Sex-based differences in immune responses are evident, with females generally showing stronger immune responses but a higher propensity for autoimmune diseases. This dimorphism is influenced by sex chromosomes, with several genes encoding immune molecules located on the X chromosome. X-linked primary immunodeficiencies (PIDs) are genetic disorders affecting the immune system, primarily observed in males due to their X-linked inheritance pattern. These disorders often present with variable clinical manifestations depending on the specific genetic mutation. Notable examples include X-linked agammaglobulinemia (XLA), X-linked severe combined immunodeficiency (XSCID), and Wiskott-Aldrich syndrome (WAS). These mutations impair the development, maturation, and function of lymphoid cells, leading to increased susceptibility to infections. Most common signs of XLA or WAS are limited to males, including absence of T cells, mature B cells, and immunoglobulins of all classes leading to severe immunodeficiency and vulnerability to pathogenic infection (157). X-linked lymphoproliferative syndrome (XLP) is typified by vulnerability to Epstein-Barr virus (EBV) infection, dysgammaglobulinemia, and lymphoma – mutations in SH2D1A and XIAP genes are implicated, affecting the immune cell functions including NK cell and T cell activities (158). X-linked primary immunodeficiencies often exhibit autoimmune manifestations, highlighting the complex interplay between immune deficiency and autoimmunity. Studies of these single-gene disorders provide insights into the pathophysiology of more complex autoimmune diseases (159).

Recent studies on X-chromosome inactivation (XCI) demonstrate that incomplete or “escape” from inactivation amplifies the dosage of immune-related genes, thus affecting disease susceptibility, clinical phenotypes, and sexual dimorphism (160–162). This emerging evidence extends beyond the classic examples of X-linked immunodeficiency syndromes, suggesting that gene dosage imbalances play a significant role in shaping female-biased autoimmunity. Anguera et al. (160) characterized large-scale transcriptional “escape” from the inactive X chromosome and revealed that key immune genes can be overexpressed in female cells. Similarly, Guery and Renaudineau (161) provide evidence that this X-linked dose effect contributes to female-biased incidence of autoimmune diseases like systemic lupus erythematosus. Chang et al. (162) extended these findings by demonstrating how partial reactivation of the inactive X can occur under inflammatory or stress conditions, further intensifying sex-based differences in immune cell function.

This dose effect is particularly relevant in diseases like lupus, where dysregulated B-cell activity and heightened interferon signaling are central. Guery and colleagues (163) demonstrated that extra X-chromosome gene expression—particularly of immune regulators—could help explain the 9:1 female-to-male ratio in lupus. In multiple sclerosis, recent XCI transcriptome analysis revealed that mosaic patterns of escape may shape T-cell exhaustion pathways, suggesting another mechanism by which females experience distinct disease courses. Overall, these newer insights emphasize that the X chromosome’s role in immunity is not merely a matter of “one copy vs. two copies” but also of epigenetic regulation, partial inactivation, and tissue-specific escape patterns. Incorporating these findings into our framework for sexual immune dimorphism provides a more nuanced view—one that moves beyond traditional X-linked deficiencies to a broader understanding of how variable XCI contributes to both protective immunity and autoimmunity in females.

## Conclusions

Sexual immune dimorphism arises from a sophisticated interplay among hormones, sex chromosomes, epigenetic mechanisms, and evolutionary processes. While sex steroids such as estrogen, progesterone, and testosterone clearly modulate immune cell function, they do so in concert with genetic and epigenetic factors—particularly partial escape from X-chromosome inactivation—that can further amplify or reduce immune responses in a sex-specific manner. Behavioral adaptations (e.g., pathogen avoidance, maternal investment strategies) also shape immune profiles, especially when considered within broader life-history and evolutionary contexts. The net outcome is a spectrum of differences in disease susceptibility and severity, with females generally exhibiting stronger immune responses—yet paying a higher cost in autoimmunity—and males showing greater vulnerability to certain infections.

Recognizing that hormonal regulation is one important facet rather than the entire story underscores the intricate crosstalk among the reproductive, immune, and endocrine systems. This comprehensive view deepens our understanding of why and how immune function diverges between males and females. It also highlights opportunities to develop more targeted, sex-specific interventions for autoimmune and infectious diseases. By integrating knowledge of hormonal influences, genetic dosage effects, behavioral ecology, and evolutionary forces, researchers and clinicians will be better equipped to design studies and treatments that respect and leverage the inherent complexities of sexual immune dimorphism. Future research directions clearly include systematic or umbrella reviews targeting narrower aspects of sexual immune dimorphism highlighted here. Such focused, quantitative syntheses would complement this narrative overview by providing detailed, exhaustive examinations of specific mechanisms or clinical outcomes.
